# Albuminuria as a Symptom of Epileptic Attacks

**Published:** 1882-09

**Authors:** 


### Albuminuria as a Symptom of Epileptic Attacks.

Kleudgen, *Arch. f. Psych.*, etc., xi, p. 478. The author concludes from an examination of 57 cases: (1) That by a certain degree of concentration traces of albumen may be detected in all urine. (2) That without increase in the specific gravity the amount of albumen may increase without our being forced to conclude that renal disease is present. (3) That post-epileptic urine has nothing characteristic about it. (4) Rarely, the post-epileptic urine is richer in albumen than before, and then only to a slight degree, and often dependent on the presence of seminal fluid. The specific gravity may be high or low after the attack; the reaction may be of an alkaline nature, or acid. Sugar was not found.—*Centralbl. f. Med. Wis.*, Oct. 22, 1881.

